# Behavioural patterns of electrolyte repletion in intensive care units: lessons from a large electronic dataset

**DOI:** 10.1038/s41598-018-30444-3

**Published:** 2018-08-09

**Authors:** Thomas T. Joseph, Matthew DiMeglio, Annmarie Huffenberger, Krzysztof Laudanski

**Affiliations:** 1 Department of Anaesthesiology and Critical Care, Perelman School of Medicine, University of Pennsylvania, Philadelphia, USA; 2 Center for Connected Medicine, University of Pennsylvania Health system, Philadelphia, USA; 3 Philadelphia College of Osteopathic Medicine, Philadelphia, USA

## Abstract

Repletion of electrolytes often depends on provider-specific behavior and hospital policy. We examined the pattern of electrolyte repletion across several intensive care units (ICU) in a large healthcare system from 2010–2015. This included 109 723 potassium repletions, 51 833 magnesium repletions, 2 306 calcium repletions, 8 770 phosphate repletions, and 3 128 249 visit-days over 332 018 visits. Potassium, magnesium, and calcium were usually repleted within the institutional reference range. In contrast, the bulk of phosphate repletion was done with pre-repletion serum level below the reference range. The impact of repletion on post-repletion levels was significant but uniformly small. The pre-repletion serum level had a significant inverse correlation with the post-repletion level of each electrolyte. Potassium, magnesium and phosphate follow-up labs were scheduled in 9–10 hours after their repletion. In contrast, calcium was rechecked in less than 20 minutes. Routine repletion of potassium, magnesium and calcium had no effect on the incidence of tachyarrhythmias. We estimated the expense from electrolyte repletion within the reference range was approximately $1.25 million. Absent a specific clinical indication, repleting electrolytes when the serum concentration are within normative values may represent an avenue for cost savings, staff burden unload and potential reduction in frequency of complications in the ICUs.

## Introduction

Repletion of electrolytes is one of the most common routine interventions during hospitalization. With respect to management of electrolyte levels in the inpatient, judicious coupling of laboratory studies with a heuristic judgment of the patient’s condition is believed to be superior to subjective clinical judgment alone^1^. Standardized protocols are often employed to improve adherence to the institutionally mandated electrolyte repletion behaviour, and, it is hoped, improve outcome^2^. Consequently, the repletion of key electrolytes could be hypothesized to follow predictable patterns. Even if good evidence exists, adherence to published guidelines is poor^3^. Several cognitive, judgment and perception biases often influence day to day clinical decision making^4–6^. The clinical benefits of these protocols are often much higher than observed, while the frequency of side effects is often underestimated. All these factors may affect how routine electrolyte abnormalities are treated, but most studies examining the patterns of electrolyte repletions were performed using small data set^7–10^.

Potassium, magnesium, calcium, and phosphate are often repleted because of a perceived need to attain a “normal” value^7^. Potassium is considered to be a critical electrolyte because of its role in maintaining optimal heart conductivity, muscle contraction, and gut motility^11^. Magnesium plays a similar role and “follows” potassium^10^. Maintaining “normal” serum levels of these electrolytes is perceived to be important despite (albeit somewhat limited) evidence that aggressive electrolyte replacement does not dramatically decrease the incidence of adverse events^9,12,13^. Furthermore, a chronically high-normal serum potassium level may be related to increased mortality^14^. Calcium and phosphate are also critical for muscle contractility^7,15,16^. Calcium boluses are sometimes used to treat hypotension, as well as certain causes of pulseless electrical activity and cardiac dysfunction^17^. Phosphate replacement is mostly employed in ICU patients with prolonged disease^7,15^.

Electrolytes have a complex, multifactorial role in homeostasis and are regulated by several interrelated mechanisms, which complicates a physiology-driven design of repletion protocols. To further underscore the confusion, it is often unclear if electrolyte abnormalities are merely symptoms or indeed are causally related to mortality^10,13^. The correlation of serum electrolyte levels to their effects is obscured by binding to protein, level of ionization, and the transmembrane electrical gradient. After all, these electrolytes are primarily located in the intracellular compartment and their biological importance depends on their ability to affect transmembrane electrical gradient^11,15,16^. Magnesium, calcium, and phosphate have a similar body distribution pattern and are vital in establishing homeostasis. Furthermore, magnesium and calcium are commonly measured as total serum concentration, even though the ionized forms are the biologically active species^18^. These are the reasons why repletion of electrolytes often fails to provide sustainable and tangible clinical effects^12^. Nevertheless, repleting electrolytes remains a mantra for providers. It is telling that implementations of protocols for aggressive repletion of electrolytes showed an improvement in satisfaction among providers and administrators, but no decrease in adverse events secondary to electrolyte abnormalities^8,12,19^. Since habits, beliefs, rituals, cognitive biases and force of inertia are inherently human features, it is understandable that these protocols may be implemented reflexively. Despite such implementation, we do not know the behavioural patterns of repletion^2,4,9,11,15^.

Here, we sought to characterize electrolyte repletion patterns in intensive care units through a large-scale, retrospective analysis. Specifically, we hypothesize that (a) the repletion threshold for all electrolytes would vary significantly from reference values, depending on individual provider preference, and (b) in many cases, repleting electrolytes would not result in clinically useful changes in corresponding serum levels. Finally, we hypothesized that electrolyte repletion practices are linked to significant economic expenses.

## Materials and Methods

The dataset used in this study was drawn from electronic medical record (EMR) data consisting of vital signs, orders, lab results, and admission dates and times for all intensive care units (ICUs) across three major hospitals in the University of Pennsylvania Health System. The data, before applying exclusions, were compiled and de-identified by institutional data warehouse services (PennOmics and Penn Data Store) before being received by the study authors. The dataset was assembled into a relational database (PostgreSQL) which was queried as necessary and statistical analysis was conducted with Python scripts using the Pandas and Statsmodels libraries.

A repletion event was defined as a lab draw, followed by administration of an electrolyte repletion, followed by another lab draw. As depicted in Fig. S1, the initial dataset included 430 137 potassium repletion events, and 238 410, 15 740, and 49 979 magnesium, calcium and phosphate repletion events respectively. The initial dataset included a total of 9 786 414 patient-days representing 458 639 visits. A visit was defined as all events and laboratory data associated with the time of transfer or admission into an ICU until discharge or death. Some patients had more than one visit; these were counted and analyzed separately.

To minimize the presence of confounders, we excluded part of the dataset (Fig. S1). We excluded patients under 18 years old (N = 50 926). We wished to exclude those visits where there was a disease process or therapeutic intervention could be expected to influence electrolyte levels, resulting in a specific clinical indication for repletion. Hence, using International Classification of Diseases (ICD-9 and -10) diagnosis codes as assigned, we excluded visits where transfusion of packed red blood cells occurred (N = 39 116), total parental nutrition orders were placed (N = 9 138), certain diagnosis codes were present: rhabdomyolysis (N = 853), a parathyroid disease of any kind (N = 642), sarcoid disease (N = 1073), and end-stage renal disease (n = 4 058). Also, visits where the patient had glomerular filtration rate (GFR) below 30 by the modification of diet in renal disease (MDRD) method were excluded as well from further analysis (n = 41 829). We note that while this and other methods for estimating GFR may not precisely reflect kidney function in the setting of acute kidney injury, it was sufficient to exclude those patients with kidney insults that may affect electrolytes. We did not, however, exclude visits of patients with chronic but not end-stage kidney disease. We also excluded “ambiguous” repletion events: those where there was more than one repletion administered between consecutive checks of the respective serum level.

The final dataset included 109 723 potassium repletions, 51 833 magnesium repletions, 2 306 calcium repletions, and 8 770 phosphate repletions. The dataset spans 332 018 visits including 3 128 249 visit-days. Of note, the number of calcium repletions included in the analysis is relatively small because we only considered those repletion events for which the clinically relevant ionized calcium level was available, since the total serum calcium level may not reflect the bio-available calcium level because of sequestration by serum albumin^16^.

Various metrics of behavioural patterns were included in the analysis: the time each order was placed (whether for electrolyte administration or a lab draw), as well as the time an electrolyte was administered and the time lab values resulted, dose of replacement, and lab results.

For our economic analysis of the impact of repletion above normative values, we utilized various public data sources. Drug prices were accessed via the Lexicomp® Online Database^20^. To adjust for slight differences in listed dosage pricing on Lexicomp® and actual dosages listed in our database, prices were scaled according to the nearest listed dosage. Lab measurement expenses were extracted from the 2017 Clinical Laboratory Fee Schedule from the Centers for Medicare and Medicaid Services (CMS)^21^. Initial IV hydration reimbursement (HCPCS Code 96360) was determined based on Physician Fee Schedule Locality for Philadelphia^22^. It is important to note that code 96360 is reported for hydration services for the initial 31 minutes to 1 hour. We chose not to include 96361 (additional hour of hydration services) to provide a more conservative estimate of cost savings. Median hourly wages for physicians, pharmacists, and registered nurses were extracted from 2016 wage estimates by the Bureau of Labor Statistics^23^. Published data was not available regarding time required to take lab measurements, place orders, and process orders. For this, we applied the following time assumptions: 5 minutes for a registered nurse (RN) to draw blood labs and execute the order, 2 minutes for the physician to place the order in the EMR, and 5 minutes for the pharmacist to process the order. These assumptions are considered conservative and likely much less than observed in clinical practice.

### Data availability

The dataset utilized in the study can be made available to readers upon request after fulfilling all institutional review board (IRB) and other regulatory requirements of the University of Pennsylvania.

### IRB

The University of Pennsylvania Perelman School of Medicine Institutional Review Board reviewed and approved the study. We were granted a waiver of consent since we used de-identified data and it was not feasible to consent several thousand patients for this retrospective study. All methods were performed in accordance with the relevant guidelines and regulations.

### Statistical Methods

Repletion thresholds were compared with the two-tailed Student t-test, as these were generally normally distributed. The Cohen d-statistic (difference in means divided by pooled standard deviation) was used to estimate effect size of chosen factors (*e.g*. patient currently on diuretics) on the repletion threshold. The ordinary least squares method was used for all linear regressions and correlations were reported as Pearson r values.

## Results

### Electrolyte repletion results in modest changes in serum levels, which depends primarily on pre-repletion serum level

We first studied the serum electrolyte level before a provider placed a repletion order. For potassium, the average such trigger across all repletions was 3.66 ± 0.36 mEq/L. Being on loop diuretics correlated to an increased pre-repletion potassium level (Table 1). Patients on potassium-sparing diuretics (spironolactone), thiazide, and acetazolamide had lower pre-repletion potassium levels. Only 34.6% of patients on diuretics of any kind received potassium replacement. Carrying a chronic kidney disease (CKD) diagnosis code was related to an increased pre-repletion serum level of potassium. Magnesium repletion followed similar trends to those in potassium. By contrast, the pre-repletion calcium level was almost identical across all studied diagnoses. In the case of phosphate only, carrying a pre-existing CKD diagnosis was related to an elevated pre-repletion serum level.

**Table 1:** Average electrolyte level before the replacement was ordered.

		Threshold	N	*p-*value	Effect size
Potassium	K_All Patients_	3.66 ± 0.36	109723		
	K_trigger off diuretics_	3.57 ± 0.31	48719		
	K_trigger on loop diuretics only_	3.75 ± 0.38	33073	≪0.00001	0.519
	K_trigger on thiazide only_	3.54 ± 0.30	6116	≪0.00001	0.098
	K_acetazolamide only_	3.54 ± 0.27	322	0.061417	0.103
	K_spironolactone only_	3.62 ± 0.44	795	0.000011	0.131
	K_CKD_	3.78 ± 0.41	6010	≪0.00001	0.578
Magnesium	Mg_All patients_	1.77 ± 0.20	51833		
	Mg_trigger off diuretics_	1.74 ± 0.19	25711		
	Mg_trigger on loop diuretics only_	1.80 ± 0.19	14257	≪0.00001	0.316
	Mg_trigger on thiazide only_	1.74 ± 0.18	2055	0.511248	0
	Mg_acetazolamide only_	1.74 ± 0.16	135	0.865312	0
	Mg_spironolactone only_	1.78 ± 0.21	531	≪0.00001	0.200
	Mg_CKD_	1.79 ± 0.22	2626	≪0.00001	0.243
Calcium	Ca_All patients_	1.05 ± 0.10	2306		
	Ca_trigger off diuretics_	1.04 ± 0.14	274		
	Ca_trigger on loop diuretics only_	1.04 ± 0.09	1479	0.843678	0
	Ca_trigger on thiazide only_	1.06 ± 0.13	19	0.665567	0.148
	Ca_acetazolamide only_	1.20 ± 0.05	3	0.053386	1.522
	Ca_spironolactone only_	1.13 ± 0.13	9	0.061671	0.666
	CA_CKD_	1.05 ± 0.07	193	0.370247	0.090
Phosphate	PO_All patients_	1.95 ± 0.58	8770		
	PO_off diuretics_	1.96 ± 0.60	4651		
	PO_loop diuretics only_	1.92 ± 0.53	2690	0.004162	0.071
	PO_trigger on thiazide only_	1.96 ± 0.60	261	0.944454	0
	PO_acetazolamide only_	1.96 ± 0.62	16	0.963447	0
	PO_spironolactone only_	2.04 ± 0.61	53	0.325411	0.132
	PO_CKD_	1.98 ± 0.51	269	0.603636	0.036

The result of the two-tailed t test is added as well and effect size is Cohen’s d statistic (difference in means divided by pooled standard deviation). The *p* value is compared to all patients that are off diuretics and do not have CKD.

**Figure 1:**
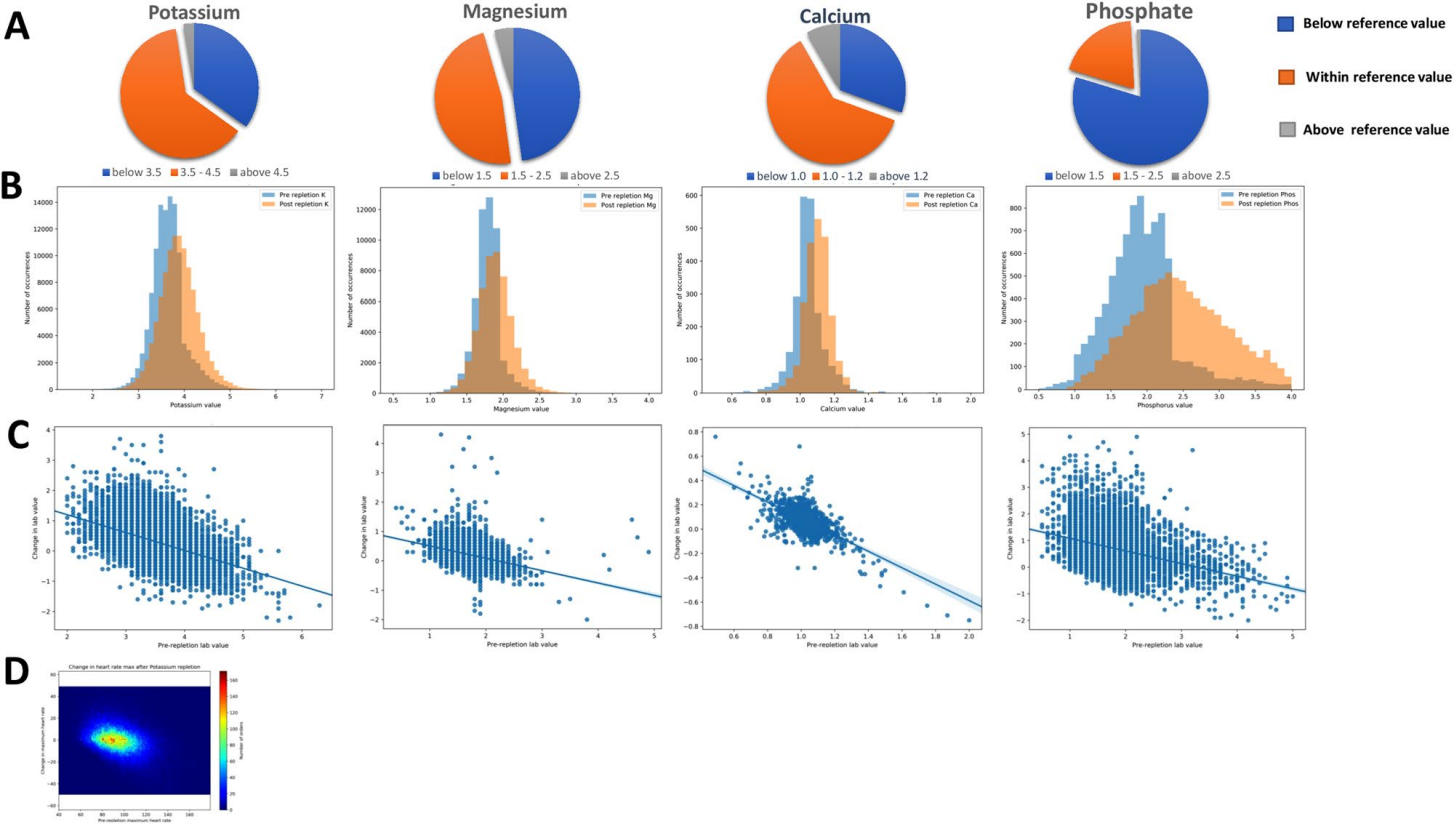
Electrolyte replacement thresholds and triggers. Majority of a replacement for potassium, magnesium, and calcium but not phosphate happened within reference ranges (**A**) and resulted in modest at best post-replacement changes in electrolytes serum level (**B**). Pre-repletion serum level correlated modestly with a post-repletion increase in lab value for all four electrolytes though these effects were relatively small (**C**). Replacement of potassium did not result in a decrease in tachyarrhythmias before and after repletion (**D**).

Most potassium, magnesium, and calcium repletions were done with pre-repletion levels above 3.5, 1.5 and 1.0 mEq/L respectively. In contrast, the serum level of phosphate was more often corrected at levels below 2.5 mEq/L (Fig. 1A).

The impact of electrolytes repletion on the serum level was significant but almost uniformly small (Fig. 1B). To measure this, we examined the serum level by lab draw which was resulted soonest after the repletion dose was recorded as given. The average change in serum values was most significant for phosphate at 0.6 mEq/L, followed by 0.26 mEq/L for potassium, 0.19 mEq/L for magnesium, and below 0.08 mEq/L for calcium (Fig. 1B). The route of administration was unimportant: for the same dose for each studied electrolyte, intravenous and oral repletions had a similar effect on the post-repletion change in electrolytes level (data not shown). Surprisingly, the most important factor correlating with the amount of increase in the post-repletion serum level of each electrolyte was the pre-repletion lab value (Fig. 1C) rather than the actual dose administered. Indeed, correlations between the dose of electrolyte given and post-repletion electrolyte serum level were significant but quite weak for potassium (r = 0.008; *t* = 5.46, *p* < 0.001), magnesium (r = 0.00079; *t* = 28.18, *p* < 0.00001) and phosphate (r = 0.005; *t* = 5.83, *p* < 0.00001). The correlation between doses of calcium given and the post-replacement level was both weak and non-significant (r = 0.000003; *t* = 0.428, *p* = 0.668).

One common reason for routine repletion of potassium is that avoiding hypokalaemia may prevent tachyarrhythmias. As a surrogate of tachyarrhythmias such as atrial fibrillation with rapid ventricular response, we examined the relationship between maximum heart rate in the 24 hours before and after a given repletion event. Potassium repletion did not result in a decrease in maximum post-repletion heart rate (Fig. 1D). A similar lack of effect was seen for magnesium and calcium (data not shown).

When evaluating repletion threshold across the three hospitals included in the dataset, clinically significant differences in repletion thresholds were not detected; all differences were within 0.5 standard deviation.

### Behaviour of providers reflected in patterns of ordering electrolyte repletion

**Figure 2:**
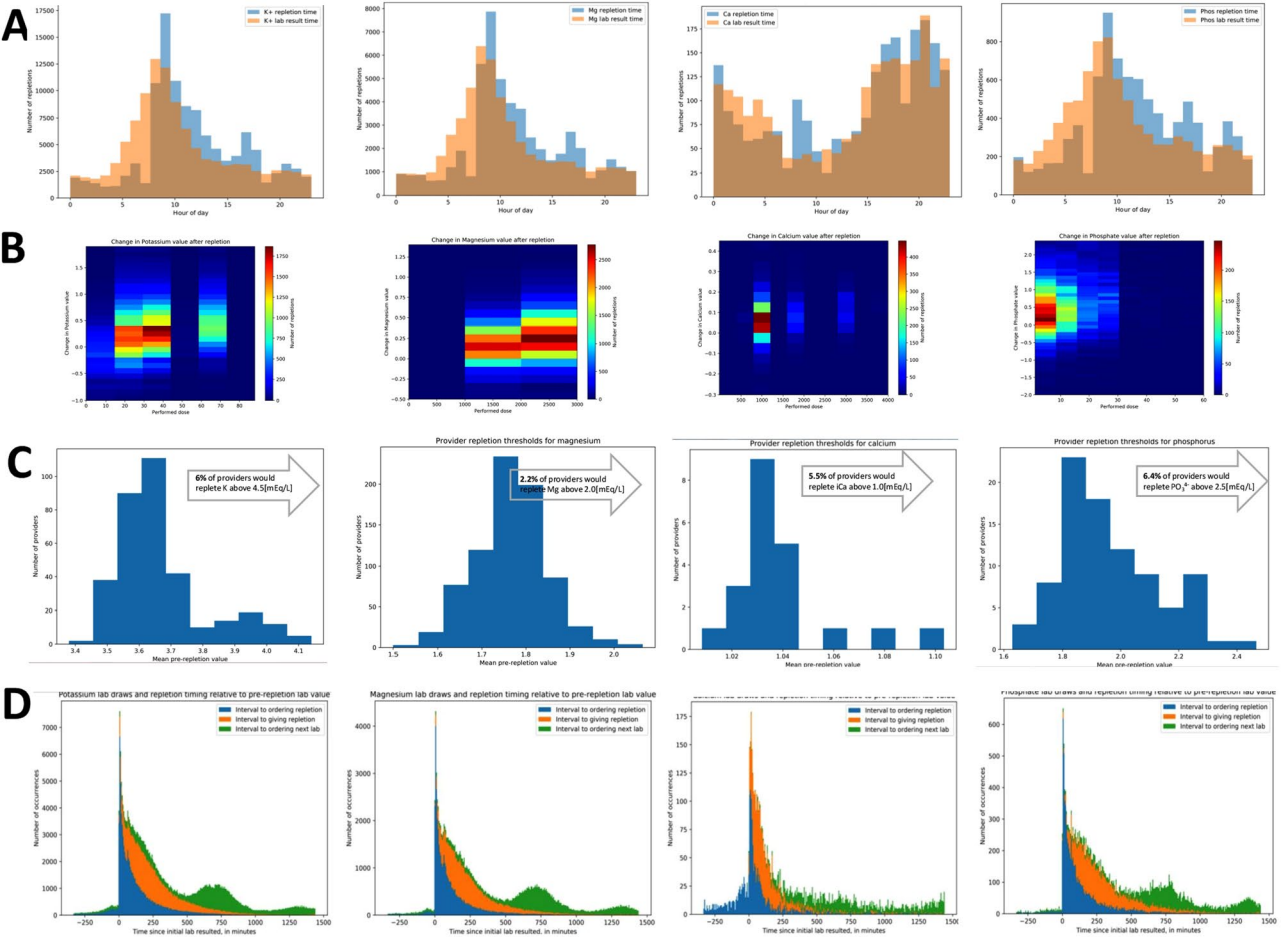
Behavioural patterns of electrolyte replacement. Majority of lab values for potassium, magnesium, and phosphate were reported in the morning with replacement orders being placed in the morning (majority) and afternoon (a significant minority) (**A**). The increased dose of electrolytes resulted only in a modest increase in post-repletion values (**B**). Small yet significant number of providers repleted electrolyte above the reference levels (**C**). The replacement timelines revealed significant overlap between placement of the order for replacement and ordering follow-up lab (**D**).

Next, we sought to examine the behaviour of providers as reflected in the patterns of repletion ordering and administration. Most lab results for potassium, magnesium, and phosphate were entered into the EMR by the laboratory in the morning around 6 AM, but this pattern was not observed for calcium (Fig. 2A). Calcium lab value were most often ordered and resulted during night hours. Notably, potassium, magnesium, and phosphate were most often repleted around 8 AM and 4 PM, while calcium repletions were more evenly spaced over a 24-hour period (Fig. 2A).

We hypothesized that the electrolyte serum level would predict the repletion dose ordered, as well as the timeliness of the order^19^. Indeed, a lower serum electrolyte level was related to a higher ordered repletion dose for all electrolytes (Fig. 2B). However, the increase in serum level was relatively small even for higher doses (Fig. 2B). As for timeliness (Table 2), the pre-repletion serum level for all electrolytes had a weak negative correlation with time to place the order (t_order_), a weak positive correlation with execution of the order by a nurse (t_RN_), and a correlation close to zero when the order was placed for follow up labs (t_order lab_).

**Table 2:** Correlation between pre-repletion lab value and time to perform certain events.

		Pre-repletion serum level		
		r	r^2^	p
Potassium	t_order_	−0.268	0.0720	≪0.0001
	t_RN_	0.273	0.0748	≪0.0001
	t_order lab_	−0.008	0.0001	0.005122
Magnesium	t_order_	−0.208	0.0434	≪0.0001
	t_RN_	0.211	0.0445	≪0.0001
	t_order lab_	0.002	~0	
Calcium	t_order_	−0.222	0.0491	≪0.0001
	t_RN_	0.231	0.0532	≪0.0001
	t_order lab_	0.052	0.0028	0.011730
Phosphate	t_order_	−0.383	0.1463	≪0.0001
	t_RN_	0.391	0.1530	≪0.0001
	t_order lab_	−0.035	0.0012	0.000940

We next asked if different providers use commonly-accepted criteria for ordering repletions – in particular, whether repletions would be ordered despite serum levels in the reference range. We computed the mean pre-repletion serum level associated with a repletion order for each provider in the dataset who had ordered at least 20 repletions, calling this the average trigger level. For potassium, 34% of providers had an average trigger level of 3.5mEq/L or greater. Similarly, 47.7% of providers had an average magnesium trigger level of 2 mEq/L or greater. 61.1% providers had an average trigger level of ionized calcium of 1 mEq/L or greater. Finally, only 19% of providers had an average trigger level for phosphate of 1.5 mEq/L or greater. Some providers placed repletion orders despite a very high serum level (Fig. 2C).

Next, we asked how long it took for providers to recheck serum levels after repletion (Fig. 2D and Table 3). Potassium, magnesium and phosphate follow-up labs were ordered on average approximately 9–10 hours after repletion, though in 15–20% of cases, followup lab orders were placed before the repletion order was actually carried out - although this does not necessarily mean that follow labs were drawn before the repletion was administered (Table 3). In contrast, calcium was rechecked less than 20 minutes after repletion.

**Table 3:** Average time to intervention from initial lab.

		Time to intervention from t_lab initial_		
		X + SD [min]	#events occurring before nominally previous event	%events occurring before nominally previous event
Potassium	t_order_	76.18 ± 297.38		
	t_RN_	279.18 ± 224.72		
	t_order lab_	696.96 ± 500.45	20073	20.3
	t_lab follow-up_	635.63 ± 443.06	0	0
Magnesium	t_order_	79.22 ± 280.32		
	t_RN_	276.17 ± 229.65		
	t_order lab_	738.05 ± 456.73	7201	15.2
	t_lab follow-up_	577.84 ± 394.91	0	0
Calcium	t_order_	−7.87 ± 309.11		
	t_RN_	176.69 ± 224.82		
	t_order lab_	856.01 ± 507.45	24	1.1
	t_lab follow-up_	19.29 ± 153.63	0	0
Phosphate	t_order_	69.21 ± 316.46		
	t_RN_	325.51 ± 242.48		
	t_order lab_	806.89 ± 501.69	1346	16.2
	t_lab follow-up_	505.33 ± 409.00	0	0

t_order_ the interval between prior lab result and placing the order.

t_RN_ interval between ordering and executing the order.

t_order lab_ the interval between completion of the replacement and ordering follow-up lab.

t_lab follow-up_ the interval between ordering the lab and follow up results showing up.

### Electrolyte repletion and kidney disease

We hypothesized that providers would be less likely to replete electrolytes in patients with kidney disease. Surprisingly, the repletion trigger for potassium and magnesium was significantly higher in patients on loop diuretics or carrying a pre-existing diagnosis of CKD (Table 1). On the other hand, the average repletion triggers for patients concomitantly taking thiazides were lower as compared to patients off diuretics, suggesting that providers are anticipating hypokalemia. At the same time, a lower potassium repletion trigger was seen in patients taking potassium sparing diuretics. Considering that this class of diuretics is known for causing potassium retention, one would expect the opposite effect. We hypothesize that providers are perhaps discounting their hyperkalemic effect. Also, most of potassium and magnesium repletion events happened when the pre-existing lab value was within reference value in our institution. Even when a very conservative trigger was used for cut-off point for potassium and magnesium was used (4.0 and 2.5 mEq/L), a significant number of replacements still occurred. Interestingly, several potassium, magnesium and phosphate replacements happened at 4 PM, which was not the peak time for any form of blood repletion. It is likely that providers are following some internal protocol, but one does not exist in the institution studied.

Repletion of calcium seems to be largely disconnected from diuretic use and occurred at a level of 1.05 mEq/L across all patients. The vast majority of replacement happened at normal levels with a scattered time pattern. Considering that ionized calcium is measured mostly in the emergency setting at our institution, we expected that most of the calcium replacement would be given during Advanced Cardiac Life Support (ACLS), acute hypotension or cardiac dysfunction^16,17^. In fact, most of these measurements were done with the help of portable, point-of-care devices suggesting that replacement occurred during an emergency. Observing a relatively high percentage of calcium gluconate versus calcium chloride replacements also supports this theory (data not shown). Nevertheless, it appears that ionized calcium levels did not influence the decision-making process of replacement.

Phosphate was repleted at 1.96 mEq/L, and the trigger was more conservative for patients carrying the code of CKD. In contrast to other electrolytes, the repletion above the normative levels was rare and focused around the time the lab value was announced in the computer system.

### Economic consequences of lab-driven replacement are significant

We hypothesized that some proportion of the interventions recorded in the dataset may be superfluous. Considering that a significant number of pre-emptive follow-up labs are placed before or at the time of electrolyte replacement, they may represent a waste (Table S1). We calculated that the expenses related to placing the order to draw the lab and the lab expense to run the sample amount to $232,846 for potassium, $66,177.20 for magnesium, and $8,749 for phosphate for the entire data set. We then calculated the total expenses related to administering electrolyte repletion above the normative values using data from Table S1. The total cost savings was $1,254,869.06 and the total time savings amounted to 343 provider-days.

## Discussion

Using a large dataset (over 300 000 entries) consisting of EMR data from multiple ICUs across three hospitals to study practices associated with routine repletion of electrolytes, we showed that providers follow haphazard patterns of care that result in significant economic consequences. Overall, it appears that potassium and magnesium are repleted routinely but with little regard to the pre-repletion serum level or effect of concurrently prescribed diuretics on the respective electrolyte levels. Calcium is repleted in total disconnect to the pre-repletion serum level. On the other hand, phosphate repletion is ordered mostly in response to abnormal serum levels. The underlying reasons for these behavioural patterns are not clear.

Maintaining “normal” levels of potassium and magnesium is believed to prevent cardiac arrhythmias and is often prioritized for patients on diuretics to avoid hypokalemia^10,16^. Conversely, patients on potassium sparing diuretic and with chronic kidney disease are perceived to be at higher risk of hyperkalaemia^11,14,16^. As electrocardiogram analyses were unfortunately not available, we addressed this question by studying whether there was a change in maximum heart rate after repletion, as a surrogate for tachyarrhythmias. No significant change in maximal heart rate was present in either group, but this assessment is of course limited. This generates the hypothesis that routine repletion of these electrolytes does not, in clinical practice, decrease the incidence of tachyarrhythmias – but answering this question would require a prospective trial.

Though the evidence base for aggressive electrolyte repletion in the absence of a specific indication is not strong, it is nevertheless considered routine practice^12,19^. Our study represent somewhat unique situation since no protocols for electrolyte replacement exist in the ICUs of three hospitals studied. Anecdotally, each of these hospitals has distinct cultural patterns which suggest a universal force driven provider behaviour. Lack of over-arching electrolyte replacement protocol leaves the providers to make decision considering their believes, habits and regional patterns^2,8,19,24,25^. Considering that the majority of repletion were given for serum levels within physiological range, we hypothesize that there is a conformity bias in decision-making processes leading to defensive repletion^2,4,11,15,16,25^.

We studied the physiological effect of repletion. All electrolytes, except phosphate, were repleted with relatively minimal effect on serum levels. Though there usually was an increase in the serum level, it correlated negatively with pre-repletion lab values and minimally with the dose. Considering that most of the follow-up labs are done in 10 to 12 hours after repletion, most of the administered electrolyte would by then be redistributed outside the intravascular compartment^26^. Furthermore, calcium and magnesium are heavily protein-bound, and only ionized forms are biologically active^15,18^. In addition, electrolyte losses are present in the healthy individual: an average adult will lose 30 to 45 mEq of potassium in urine daily which is normally replaced by normal oral intake. If the patient is kept *nil per os* (NPO) this will result in depletion of potassium stores. If the patient is on a non-potassium-sparing diuretic this is exacerbated^11,13,16^. Consequently, the prescribed repletion doses of potassium may barely even cover the losses, let alone replete the stores.

This study offers insight into the time dynamics behind electrolyte replacement. We observed a weak correlation between pre-repletion serum level and time to place the order, but these correlations disappear as the next step of electrolyte repletion occurred^16^. One could anticipate that more severe form of electrolyte depletion would trigger vigorous and more robust response from providers but this was not the case: correlations between given doses, the time to begin infusion and pre-intervention lab value were weak. We believe that large variability in practice patterns exists and are related to several biases in clinical decision making^2,4,5,25^. Even more interestingly, we showed that overlaps occurred in some cases between the timing of the pre-repletion lab result, initiation of the repletion infusion, and timing of the follow-up lab order This behaviour is not unprecedented and represent a provider pre-emptively taking the next step - a sign of routine care^14,19,24,27^. It is also possible that critical care nurses anticipate orders by providers and do the task before they are asked or before the order is entered since these events could be charted when they were entered into the computer system and not when they were actually performed. Alternatively, a significant number of orders may have been initially made via. informal verbal orders, but that route is strongly discouraged in our institution. Overall, the pattern follows the hospital “circadian rhythm” for magnesium and potassium – that is, it coincides with shift changes and the usual times of morning and afternoon rounds further suggesting informal patterns in the electrolyte replacement practices^9,19^. In contrast, whether the day of the week for a given repletion event was a weekday (Monday through Friday) or a weekend (Saturday or Sunday) did not have a clinically significant effect on the distribution of times to repletion, nor on the electrolyte trigger level.

Finally, one has to take into account the impact of superfluous electrolyte replacement^11,14^. We used two models to calculate the financial expense of electrolyte replacement to calculate the workforce and economic consequences^21–23^. We uncovered significant potential savings, even after applying exclusion criteria resulting in more than 70% of our database records being eliminated. Although the model can be disputed in the assigned monetary value, we believe that substantial room for improvement exists. In addition, saving the time and effort of providers and nursing frees them to do other tasks and could result in decreased burnout^28,29^. It may also reduce from central line infection or complications from placement or phlebitis by reducing the usage of central lines^30^. A small but significant proportion of repletion were done despite a serum level over the upper reference limit. Less than 2% of potassium repletions were given in the setting of a serum potassium level above 4.5 mEq/L. There is no obvious indication to replete potassium in this situation, so we assume these were erroneous in some way. Potentially, the lab value was not accessible or not considered by the provider. However, repletion of electrolyte on super-physiological level represent potential for severe adverse effects and can be considered a sentinel event.

The dataset studied here, while curated, has some limitations. The large magnitude of the intercepts on regression analysis, as well as in many cases relatively low R^2^ values, suggests the existence of other factors affecting electrolytes changes. Our data set is subject to the typical limitations of EMR data including errors related to inaccurate objective data entry, verbal orders not being correctly entered, or back charting. It is also our assumption that electrolyte level before repletion was a trigger for the repletion. This assumption may be challenged and is probably not valid in case of majority of calcium replacement. While the reasons for this are unclear, this potentially places patients at risk for co-morbidities secondary to excessive electrolyte levels.

Despite these limitations, we found that superfluous replacement of electrolytes was widespread. It is likely that the local hospital culture, cognitive biases and other forces influences provider behaviour even if a guideline or hospital protocol would state otherwise. Over the course of the study period, curtailing such electrolyte replacement behaviour would have resulted in $1.25 million in savings on the costs of the medications, laboratory measurements, and provider and nursing time spent. Such goal could be accomplished by introduction of protocol designed with cognitive biases of providers in mind.

## Conclusion

In a retrospective analysis of EMR data from multiple hospitals in the same health system, we found that the patterns of routine electrolyte repletion in intensive care units in our health system, in the absence of specific clinical indications, followed unexpected patterns that do not uniformly fit with established medical reasoning. We suggest that eliminating these repletion behaviours could result in significant time and monetary saving.

## Electronic supplementary material

Supplementary Table S1. Cost analysis of the electrolyte replacement in case of electrolytes repleted above reference limit.

Supplementary Figure 1. Dataset and exclusions criteria reported in accordance with EQUATOR NETWORK
