# Supplementary material for: Behavioural patterns of electrolyte repletion in intensive care units: lessons from a large electronic dataset
Source: Sci Rep. 2018 Aug 9;8:11915. doi: 10.1038/s41598-018-30444-3 (PMC6085366; doi:10.1038/s41598-018-30444-3)
Supplement: Supplementary file 1 — Supplementary Table S1. Cost analysis of the electrolyte replacement in case of electrolytes repleted above reference limit. [file 41598_2018_30444_MOESM1_ESM.docx]

# Behavioural patterns of electrolyte repletion in intensive care units: lessons from a large electronic dataset

**Supplementary Table**

Thomas T. Joseph^1^, Ann Huffenberger^2^, Matthew DiMeglio^3^, Krzysztof Laudanski^1^

^1^Department of Anaesthesiology and Critical Care, Perelman School of Medicine, University of Pennsylvania,

^2^ Penn eLert, University of Pennsylvania

^3^ Philadelphia College of Osteopathic Medicine


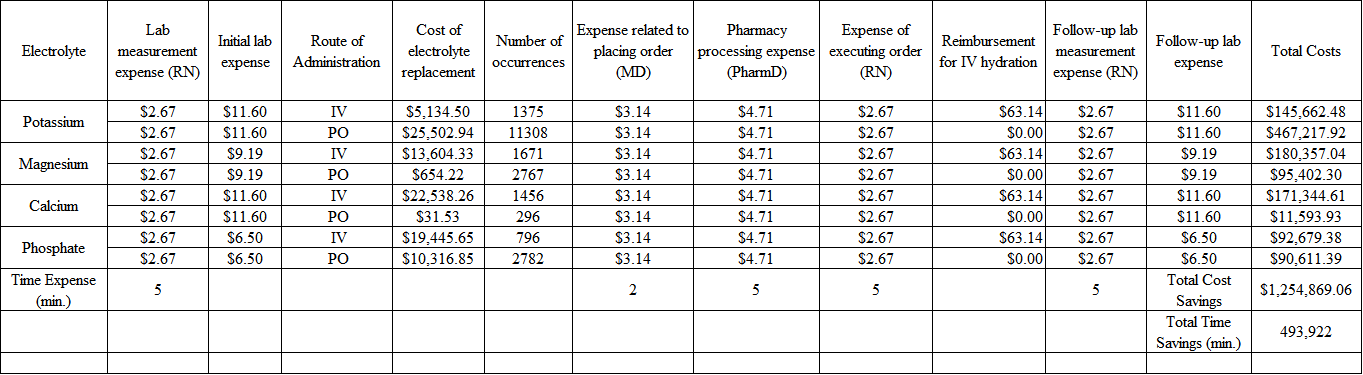


**Supplementary Table S1. Cost analysis of the electrolyte replacement in case of electrolytes repleted above reference limit.**
